# Integration of transcriptome and targeted metabolome profiling reveals hormone related genes involved in the growth of *Bletilla striata*

**DOI:** 10.1038/s41598-021-01532-8

**Published:** 2021-11-09

**Authors:** Hengwei Zou, Hanxiao Jiang, Liangbo Li, Rongshao Huang

**Affiliations:** College of Agriculture, Guangxi University, Nanning, 530005 China

**Keywords:** Biochemistry, Molecular biology, Plant sciences

## Abstract

*Bletilla striata* (Thunb.) Reichb.f. (BS) is a traditional Chinese medicine with numerous beneficial effects. In our previous study, *Aspergillus flavus* was isolated from *B. striata*. To explore the physiological and molecular mechanisms of *Aspergillus flavus* elicitor (1-G4) that promoted *Bletilla striata* growth, in this study, we performed the determination of growth indexes and transcriptomics and metabolomics analysis under 5% and 10% 1-G4 conditions. Results showed that 1-G4 elicitor could significantly promote the growth and development of *B. striata*. With the increasing concentration of 1-G4 elicitor, the contents of SA, ICAld, and ME-IAA significantly increased while the IP and ACC contents decreased dramatically. A total of 1657 DEGs (763 up-regulated and 894 down-regulated) between the control (CK) and 5% elicitor (CK vs G5) and 2415 DEGs (1208 up-regulated and 1207 down-regulated) between the control and 10% elicitor (CK vs G10) were identified. Further, we found that 22, 38, and 2 unigenes were involved in ME-IAA, IP, and ACC, respectively. It was indicated that these unigenes might be involved in *B. striata* growth. Overall, the current study laid a theoretical foundation for the effective utilization of endophytic fungi and the optimization of germplasm resources of *B. striata*.

## Introduction

*Bletilla striata* (Thunb.) Reichb.f. (BS) is an endangered orchid with high ornamental and medicinal values^1^ and likes warm and humid climates, mainly distributing in Guizhou, Sichuan, Hunan, Hubei, Anhui, Henan, Zhejiang, Shanxi, etc., in China. The dry tuber of *B. striata* is traditional Chinese medicine, that has a history of thousands of years and is widely used in Eastern Asian countries with numerous beneficial effects in the treatment of gastrointestinal mucosal injury, ulcer, bleeding, bruise and burn^1, 2^.

In the long-term coordinated evolution process, the fungi successfully destroyed the host plant's defense systems and colonized the intercellular space of the host plants. To adapt to the complex ecological environment, host plants have developed a fungal adaptation mechanism^3, 4^. On the one hand, host plants provide nutrients (photosynthetic products and mineral elements) for the growth and development of endophytic fungi; On the other hand, endophytic fungi can produce active metabolites, including extracellular enzymes, plant hormones, and active compounds. These metabolites can promote the growth and development of host plants, enhance the resistance of host plants to biotic and abiotic stresses or increase the accumulation of metabolites of host plants^5^.

At present, research on *B. striata* mainly focuses on chemical pharmacology, tissue culture, and the introduction and domestication of plants^6, 7^, however, there are few studies elaborating on the mechanisms of promoting the growth of endophytic fungi in the *B. striata*. In our previous study, 24 strains of endophytic fungi were isolated from the roots and tubers of *B. striata*. One of the strains was identified as *Aspergillus flavus,* named as 1-G4, which could promote the growth of *B. striata* in elicitor culture. Therefore, the present study aimed to explore the physiological and molecular mechanisms of 1-G4 promoting the growth of *B. striata* through transcriptomics and metabolomics methods, to lay a theoretical foundation for the effective utilization of endophytic fungi and the optimization of germplasm resources of *B. striata*.

## Materials and methods

### Medium configuration of 1-G4 elicitor

The 1-G4 fermentation homogenate was broken by ultrasonic wave, filtered, and sterilized to produce 1-G4 elicitor solution (pure elicitors). Then the elicitors were diluted into 10% and 50% elicitor solutions with potato dextrose broth (PDB) and added into MS medium with pure elicitors in a ratio of 1:9 to finally prepare 5% and 10% elicitor medium, named as G5 and G10, respectively.

### Experimental materials

The seedlings of *B. striata* were purchased from Xinxin Biotechnology Co., Ltd (Pu'an County, Guizhou Province, China) and were identified by Prof. Rongshao Huang (Guangxi university). The collection of plant materials complies with relevant institutional, national, and international guidelines and legislation. The seedlings along with the same size of corm were taken out, the roots were removed, and only part of the main stem was retained as the research material. The density of 4 plants/bottle was used to inoculate into the inducer medium, and a medium containing only the same volume of PDB was used as a control (CK). After 60 days, measurement of the morphological data of *B. striata* was performed with three plants, then we took the average. The fresh corms were collected and used for transcriptome and hormone metabolism analysis.

### RNA library preparation for transcriptome sequencing

From each sample, we used a total of 1.5 µg RNA as input material for RNA sample preparation. Following the manufacturer’s recommendations, the NEBNext Ultra RNA library preparation kit for illumina (NEB, USA) was employed to generate sequencing libraries, and add an index code to assign the sequence to each sample. In short, the mRNA was purified from the total RNA using poly-Toligo-attached magnetic beads. The NEBNext first-strand synthesis reaction buffer (5×) with high-temperature divalent cations was used for fragmentation. The synthesizing of the first-strand cDNA was done using random hexamer primers and M-MuLV reverse transcriptase (RNase H-). Subsequently, DNA polymerase I and RNase H were used for second-strand cDNA synthesis. The residual overhangs were transformed to blunt ends by exonuclease/polymerase activity. After the 3' ends of the DNA fragments were adenylated, the NEBNext adaptor having a hairpin loop structure was ligated for hybridization. To select cDNA fragments with a length of preferably 250–300 bp in length, and AMPure XP system was employed to purify the library fragments (Beckman Coulter, Beverly, USA). Then, a total of 3 µl USER Enzyme (NEB, USA) and the size-selected, adaptor-ligated cDNA were used at 37 °C for 15 min, then at 95 °C for 5 min, and then PCR was performed. Then PCR was carried out with Phusion High-Fidelity DNA polymerase, Universal PCR primers, and Index (X) Primer. Finally, the PCR products were purified (AMPure XP system) and the library quality was evaluated on the Agilent Bioanalyzer 2100 system.

### Transcriptome assembly and gene functional annotation

The assembly of the transcriptome was performed using Trinity^8^, where min_kmer_cov, was set to 2 by default, and entire other parameters were set to default. Gene functional annotation was conducted based on the following databases: national center for biotechnology information (NCBI) non-redundant protein sequences (Nr), NCBI non-redundant nucleotide sequences (Nt), clusters of orthologous groups of proteins databases (KOG/COG), protein family (Pfam), a manually annotated and reviewed protein sequence database (Swiss-Prot), gene ontology (GO), and KEGG ortholog database (KO).

### Differential gene expression analysis

A DESeq2 R package (1.20.0) was used to obtained differential gene expression analyses. DESeq2 implement a statistical approach to define differential expression in digital gene expression data via a model built on the negative binomial distribution. The resulting *p* values were adjusted using Benjamini and Hochberg’s method to control the false discovery rate^9^. A gene was considered differentially expressed if at *p* value created by DESeq2 was < 0.05.

### Functional enrichment analysis of differentially expressed genes (DEGs)

The clusterProfiler R package, with gene length bias correction, was executed for enrichment analysis of DEGs through GO database. A corrected *p* value > 0.05 for GO terms means differentially expressed genes were significantly enriched. The kyoto encyclopedia of genes and genomes (KEGG) serves as a database resource to establish an understanding of advanced-level gene functioning and utilities of the genes in biological systems (such as the cell, the organism, and the ecosystem), from information at the molecular level (especially large-scale molecular) datasets generated by genome sequencing and other high-throughput experimental technologies (http://www.genome.jp/kegg/). The statistical enrichment analyses of DEGs in the KEGG pathways were carried out via clusterProfiler R package.

### Sample preparation and extraction for LC–MS/MS

For sampling preparation, fresh leaves were collected, weighted, and quickly frozen in liquid nitrogen and were kept at − 80 °C until needed. An approximate 50 mg of leaves (fresh weight) were ground into powder by liquid nitrogen and extracted through methanol/water/formic acid (15:4:1, V/V/V). The evaporation of combined extracts was performed till dryness under the nitrogen gas stream, reconstituted in 80% methanol (V/V), and filtrated (PTFE, 0.22 μm; Anpel) before LC–MS/MS analysis.

### HPLC conditions

The sample extracts were analyzed using an LC–ESI–MS/MS system (HPLC, Shim-pack UFLC SHIMADZU CBM30A system; MS, AppliedBiosystems Triple Quadrupole 6500). The analysis was performed as described in Kasote et al.^10^ with some modifications. HPLC: column, Waters ACQUITY UPLC HSS T3 C18 (1.8 µm, 2.1 mm*100 mm); temperature: 40 °C; solvent system, water (0.05% acetic acid): acetonitrile (0.05% acetic acid); flow rate: 0.35 ml/min; injection volume: 2 μl; gradient program: 95:5 V/V at 0 min, 95:5 V/V at 1 min, 5:95 V/V at 8 min, 5:95 V/V at 9 min, 95:5 V/V at 9.1 min, 95:5 V/V at 12 min. The effluent was alternatively connected to an ESI-triple quadrupole-linear ion trap (QTRAP)-MS.

### ESI-QTRAP-MS/MS

We used AB 6500 QTRAP LC/MS/MS System, which was equipped with an ESI Turbo Ion-Spray interface and controlled by Analyst 1.6 software (AB Sciex). The operation parameters were as follows: an ion source: turbo spray; ion spray voltage (IS): 4500 V; source temperature: 500 °C; the collision gas (CAD) was medium and curtain gas (CUR) was set at 35.0 psi. CE and DP for specific MRM transitions were conducted with further CE and DP optimization. An explicit set of MRM transitions was monitored for each period based on the plant hormones eluted within this period.

### Detection of phytohormones

Based on the AB Sciex QTRAP 6500 LC–MS/MS platform, the contents of phytohormones were detected by MetWare, Wuhan, China (http://www.metware.cn/). Each assay was performed in three repeats.

## Results

### Effects of 1-G4 elicitor on *B. striata* growth and development

**Figure 1:**
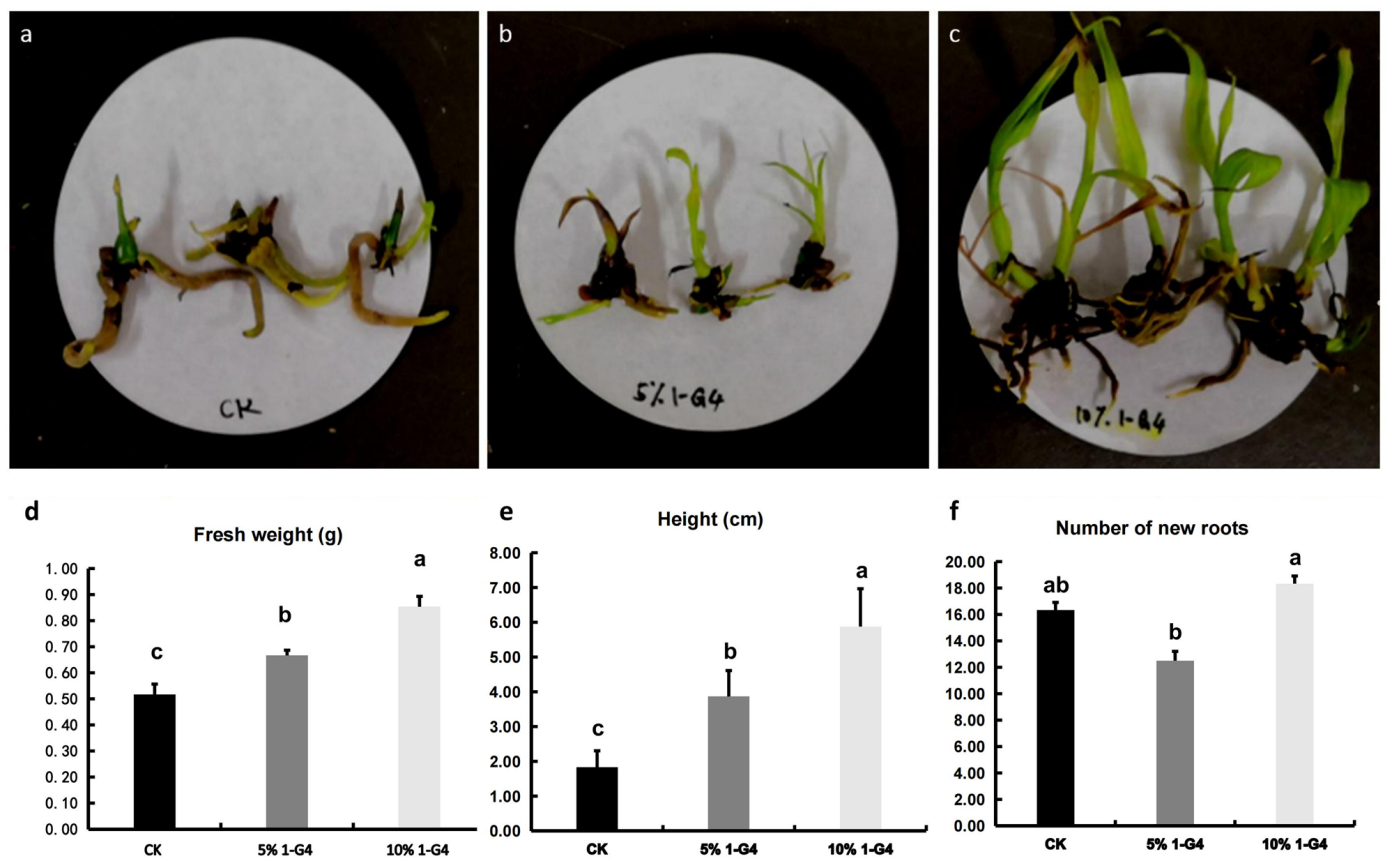
The growth and development of *B. striata* under different concentrations of 1-G4 elicitor. (**a**–**c**) Phenotypic changes of control, 5% 1-G4, and 10% 1-G4 treated BS. (**d**) Fresh weight of control, 5% 1-G4, and 10% 1-G4 treated BS. (**e**) Height of control, 5% 1-G4, and 10% 1-G4 treated BS. (**f**) New roots number of control, 5% 1-G4, and 10% 1-G4 treated BS.

In this study, 5% and 10% 1-G4 elicitors were applied on *B. striata*. Results showed that 10% 1-G4 elicitor could significantly promote the growth and development of *B. striata* (Fig. 1a–c). With the increase of 1-G4 elicitor concentration, the fresh weight and plant height significantly increased, respectively (Fig. 1d,e). Under the 5% 1-G4 elicitor condition, the fresh weight and plant height increased by 28.85% and 111.48%, compared with the control. When the concentration increased to 10%, the fresh weight and plant height increased by 63.46% and 221.31%, respectively. In addition, the number of new roots was greatly reduced under the 5% 1-G4 elicitor condition, in comparison with the 10% 1-G4 (Fig. 1f).

### Hormonal changes under different concentrations of 1-G4 elicitor

**Figure 2:**
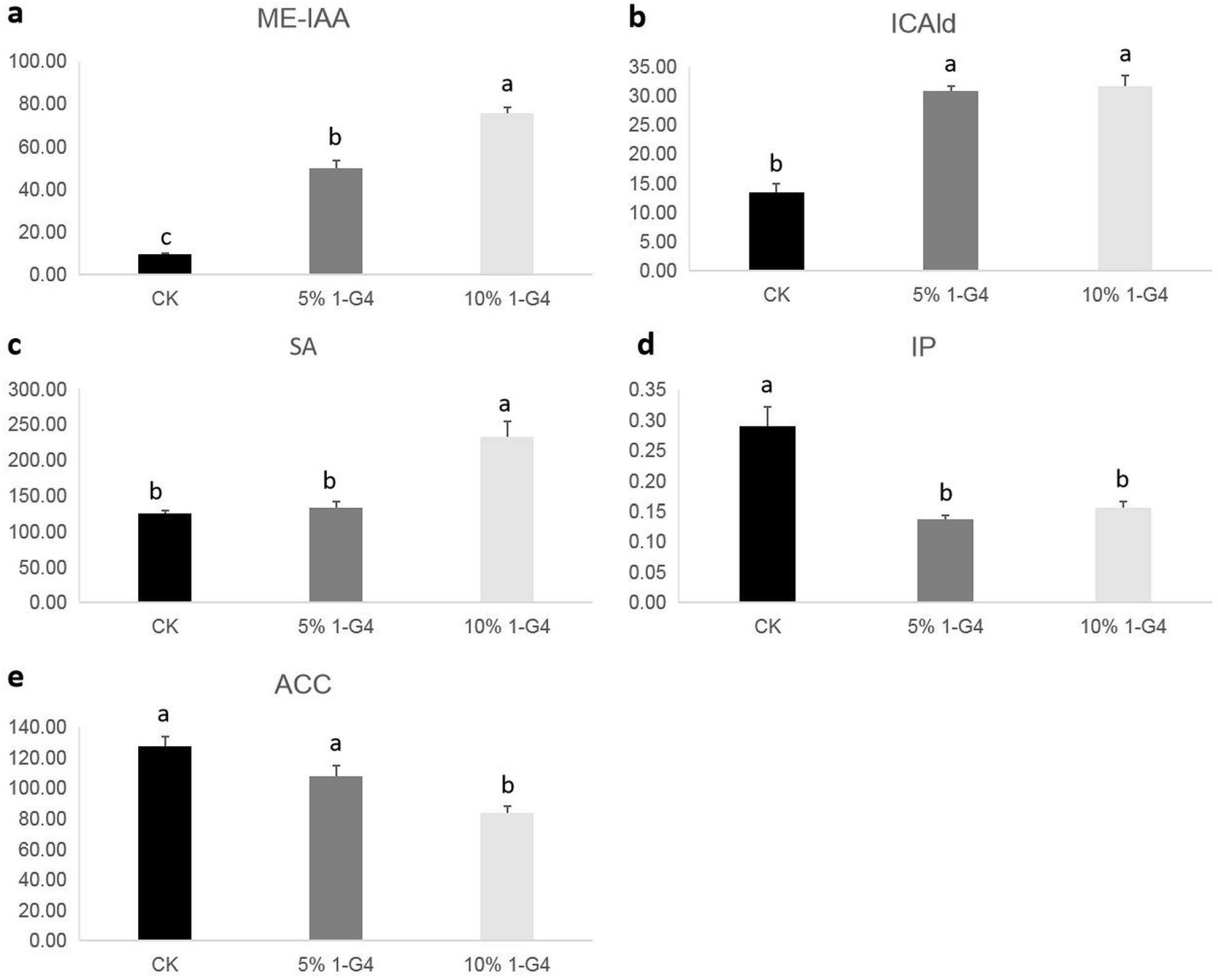
Changes in hormones under different concentrations of 1-G4 elicitor. (**a**) ME-IAA levels in control, 5% 1-G4 and 10% 1-G4 treated BS. (**b**) ICAld levels in control, 5% 1-G4 and 10% 1-G4 treated BS. (**c**) SA levels in control, 5% 1-G4 and 10% 1-G4 treated BS. (**d**) IP levels in control, 5% 1-G4 and 10% 1-G4 treated BS. (**e**) ACC levels in control, 5% 1-G4 and 10% 1-G4 treated BS.

In the present study, to understands the relationship between hormones and the growth and development of *B. striata*, the hormone metabonomics was performed (Fig. 2). Results showed that with the increasing concentration of 1-G4 elicitor, the content of SA, ICAld, and ME-IAA significantly increased. Compared with the control, SA, ICAld, and ME-IAA content increased by 5.84%, 128.96%, and 422.36% under the 5% 1-G4 elicitor condition and increased by 85.68%, 134.41%, and 692.45% under 10% 1-G4 elicitor condition, respectively. Conversely, the IP and ACC contents decreased dramatically under the 5% and 10% 1-G4 elicitor conditions. The contents of IP and ACC were reduced by 52.93% and 15.51% under 5% 1-G4 condition, and those were reduced by 46.04% and 34.33% under 10% 1-G4 elicitor condition, respectively.

### RNA sequencing, *de novo* assembly, and functional annotation

In this study, we performed RNAseq of the 9 samples using the Illumina Nova seq platform (Table S1). The datasets were available in the NCBI repository (PRJNA732250). Results showed that a total of 424,504,974 bp of raw reads and 414,881,176 bp of clean reads were generated. For each sample, the Q20 and Q30 were greater than 97% and 93%, respectively. The error rate was less than 0.03%, and the GC content ranged from 45.56 to 46.81%. Based on the high quality of sequencing data, the transcripts and unigenes were functionally annotated. In total, 193,003 transcripts were identified (Table S2, Fig. S1), including 58,245, 47,084, 40,697, and 46,977 transcripts with long intervals of 301–500 bp, 501–1000 bp, 1001–2000 bp, and > 2000 bp, respectively. Then a total of 95,657 unigenes were annotated against NR, GO, KEGG, eggNOG, Swiss-Prot, and Pfam databases, including 35,101, 29,307, 16,164, and 15,085 unigenes with a length range of 301–500 bp, 501–1000 bp, 1001–2000 bp, and > 2000 bp, respectively (Table S3, Fig. S1). Among those, 41,532 unigenes, accounting for 43.41% of the total unigenes were annotated to the NR database, while 24,375 (25.48%), 9168 (9.58%), 24,382 (25.48%), 6027 (6.3%), and 23,559 (24.62%) unigenes could be annotated to GO, KEGG, Pfam, KOG, and Swissport databases, respectively (Fig. S2).

**Figure 3:**
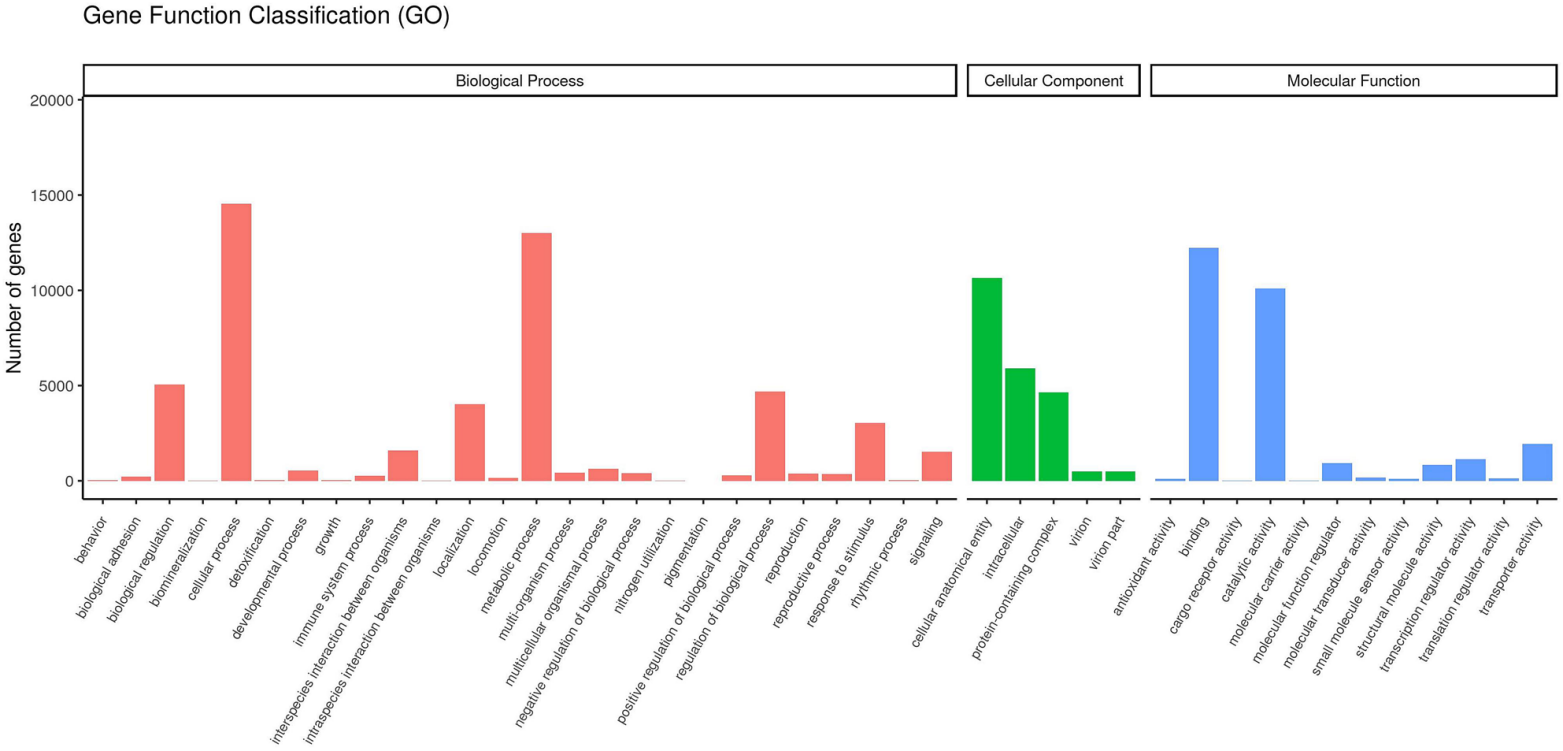
GO categories of the assembled unigenes. X-axis represents the GO terms names; Y-axis represents the number of unigenes: red pillars represent biological process, green pillars represent cellular process, and blue pillars represent molecular function.

Gene Ontology (GO) analysis showed that those unigenes were distributed under three major GO categories which included biological process (BP), cellular component (CC), and molecular function (MF) term (Fig. 3). For biological process, metabolic process (13,011 unigenes) and cellular process (14,553 unigenes) were the most significantly enriched terms; under the cellular component, cellular anatomical entity (10,653 unigenes) was the most significant enrichment; under molecular function, binding (12,224 unigenes) and catalytic activity (10,087 unigenes) were the most significantly enriched terms.

**Figure 4:**
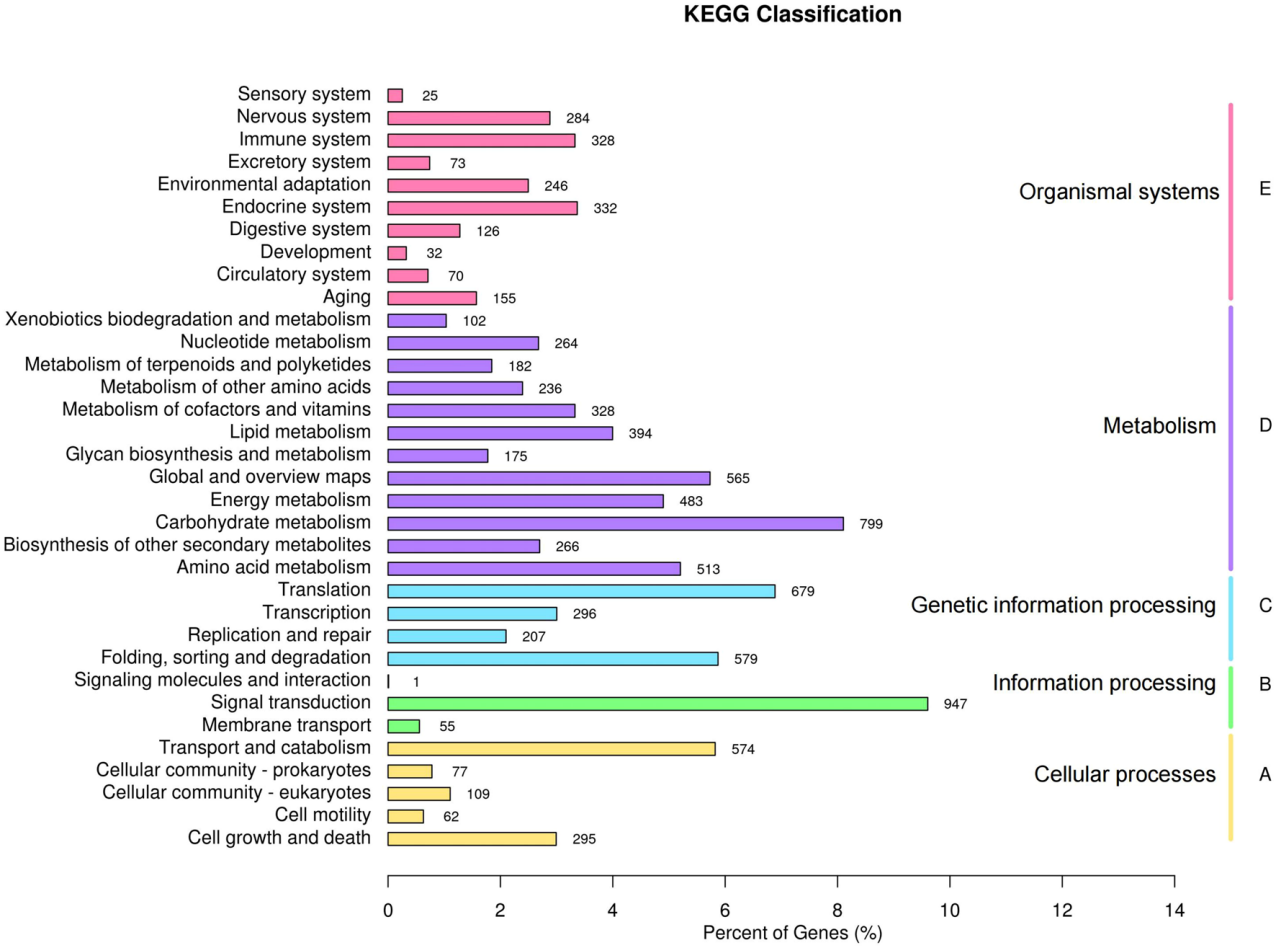
KEGG metabolism pathway categories of the assembled unigenes. X-axis represents the percentage of unigenes; Y-axis represents the KEGG classification: pink pillars represent organismal systems, purple pillars represent metabolism, cyan pillars represent genetic information processing, green pillars represent information processing and yellow pillars represent cellular processes.

Further, we obtained the active biological functional pathways on *B. striata* corm unigenes from the KEGG pathway database (Fig. 4). A total of 9859 unigenes were aligned with 34 classifications and the pathways were classified into five categories, including cellular processes (A), environmental information processing (B), genetic information processing (C), metabolism (D), and organismal systems (E). Of the 34 KEGG categories, the cluster for signal transduction (947) represented the largest group, followed by carbohydrate metabolism (799) and translation (679). The metabolism pathways were classified into 12 branching routes including carbohydrate metabolism, energy metabolism, biosynthesis of other secondary metabolites, amino acid metabolism, lipid metabolism, and glycan biosynthesis and metabolism, and so on.

### Analysis of differentially expressed genes (DEGs)

**Figure 5:**
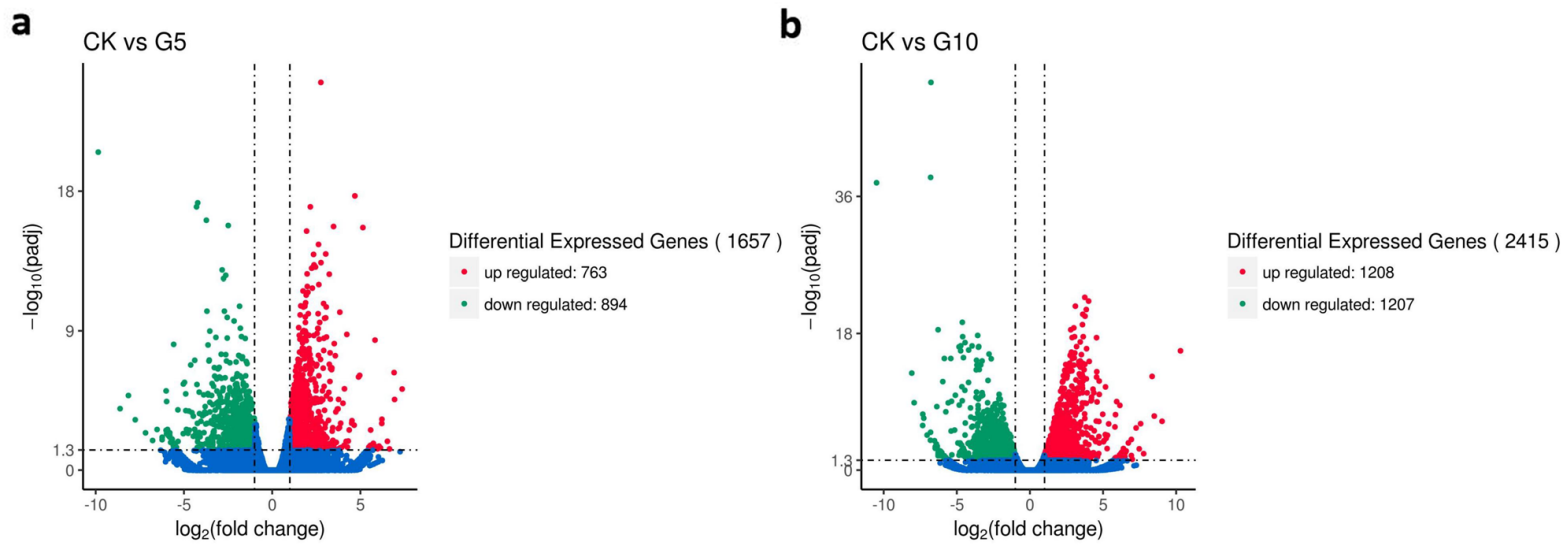
Comparison of DEGs in CK versus G5 and CK versus G10. (**a**) The differential expressed genes between CK and 5% 1-G4 treated BS. (**b**) The differential expressed genes between CK and 10% 1-G4 treated BS. X-axis represents the value of − log_10_ (padj); Y-axis represents the value of log_2_ (fold change): green spots represent down-regulated genes and the red spots represent the up-regulated genes.

To identify the genes involved in the growth and development of *B. striata* under different 1-G4 elicitor levels, we analyzed the DEGs among the treatments using the following parameters: *p* value < 0.05 and |log_2_ FC (fold change)|≥ 1. A total of 1657 DEGs with 763 up-regulated (red dots) and 894 down-regulated genes (green dots) between control and 5% elicitor medium (CK vs G5) (Fig. 5a), and 2415 DEGs with 1208 up-regulated and 1207 down-regulated genes between control and 10% elicitor medium (CK vs G10) were obtained (Fig. 5b).

**Figure 6:**
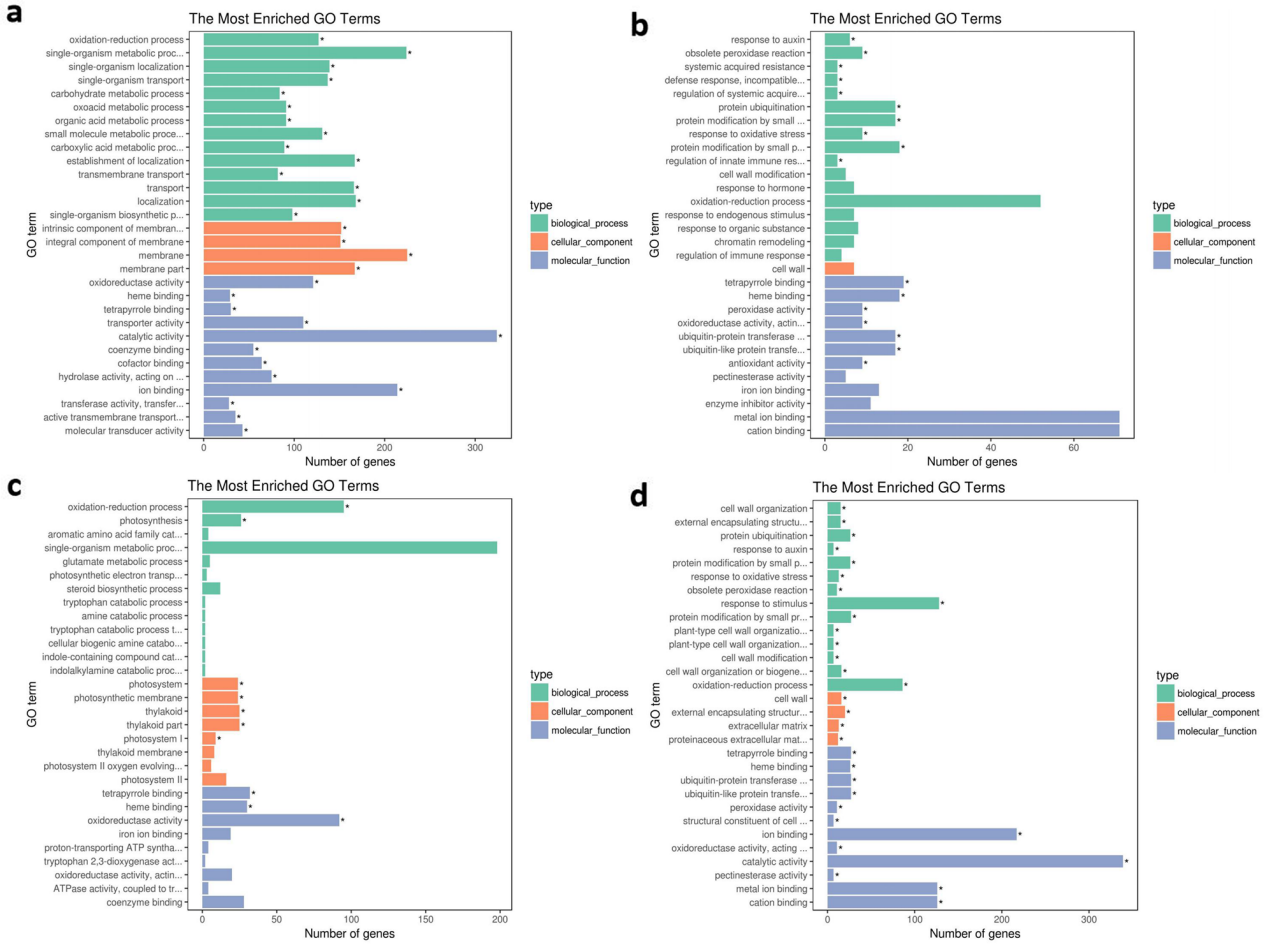
GO enrichment analysis of the DEGs in CK versus G5 and CK versus G10. (**a**) Down-regulated unigenes in control and 5% 1-G4 treated BS. (**b**) Up-regulated unigenes in control and 5% 1-G4 treated BS. (**c**) Down-regulated unigenes in control and 10% 1-G4 treated BS. (**d**) Up-regulated unigenes in control and 10% 1-G4 treated BS. X-axis represents the number of unigenes; Y-axis represents the GO terms names: green pillars represent biological process, orange pillars represent cellular process and blue pillars represent molecular function.

GO enrichment analysis revealed that the DEGs belonged to certain molecular functions (MF), biological processes (BP), and cellular components (CC) (Fig. 6). For CK vs G5, a total of 2682 GO pathways involved in all the GO terms of MF, BP, and CC categories were significantly down-regulated in comparison with control (Fig. 6a), while 1517 GO pathways were involved in more than half of the GO terms of MF and BP categories were found significantly up-regulated (Fig. 6b). In CK vs G10, 2050 GO terms including oxidation–reduction process and photosynthesis in BP, photosystem, photosynthetic membrane, thylakoid, thylakoid part, and photosystem I in CC, and tetrapyrrole binding, heme binding, and oxidoreductase activity in MF category were revealed dramatically down-regulated (Fig. 6c), conversely, all the 1898 GO terms presented significant up-regulated (Fig. 6d). Therefore, it could be seen that the increase in the concentration of 1-G4 enabled a dramatic decrease in the number of significantly down-regulated GO terms, while facilitated a remarkable increase in the number of significantly up-regulated GO terms.

**Figure 7:**
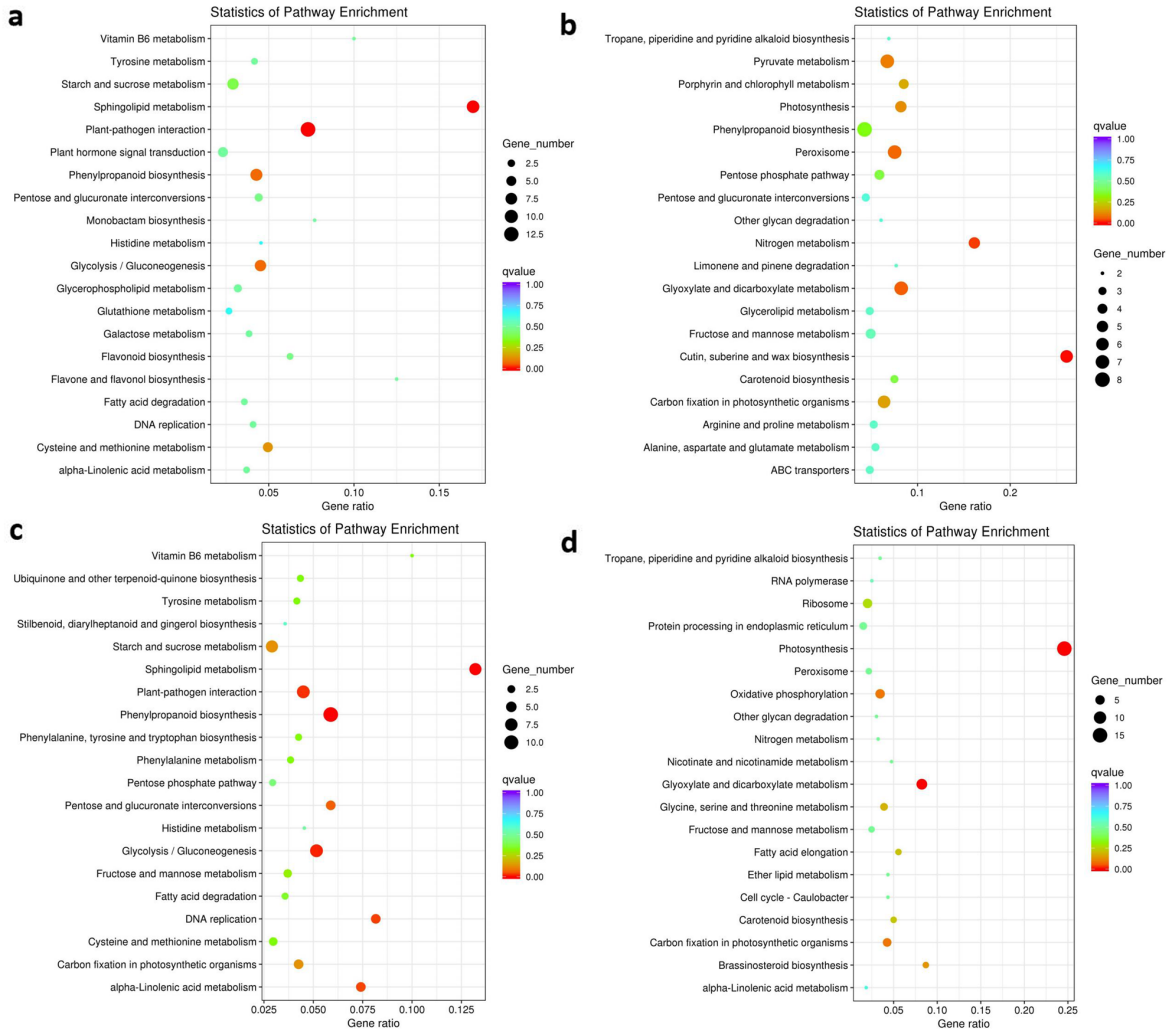
KEGG enrichment analysis of the DEGs in CK versus G5 and CK versus G10. (**a**) Down-regulated unigenes in control and 5% 1-G4 treated BS. (**b**) Up-regulated unigenes in control and 5% 1-G4 treated BS. (**c**) Down-regulated unigenes in control and 10% 1-G4 treated BS. (**d**) Up-regulated unigenes in control and 10% 1-G4 treated BS. X-axis represents the gene ratio; Y-axis represents the KEGG pathway, the size of spots represents the number of gene, and the color of spots represents the q-value of enrichment.

The KEGG analysis revealed that a total of 87 and 59 KEGG pathways were involved in CK vs G5 and CK vs G10, respectively. As shown in Fig. 7, the top 20 up- and down-regulated KEGG pathways were identified in CK vs G5 and CK vs G10. Among these, 78 down-regulated and 87 up-regulated unigenes were identified in CK vs G5, while 78 down-regulated and 60 up-regulated unigenes were detected in CK vs G10. As the concentration of 1-G4 elicitor increased, the significantly up-regulated pathways included photosynthesis, oxidative phosphorylation, ribosome, protein processing in the endoplasmic reticulum, and glycine, serine and threonine metabolism, etc., and the dramatically up-regulated pathways contained phenylpropanoid biosynthesis, plant-pathogen interaction, DNA replication, carbon fixation in photosynthetic organisms, fructose and mannose metabolism, and cysteine and methionine metabolism, etc. It was inferred that those pathways might play a vital role in the growth and development of the *B. striata*.

### Integrative analysis of hormone and transcriptome

**Figure 8:**
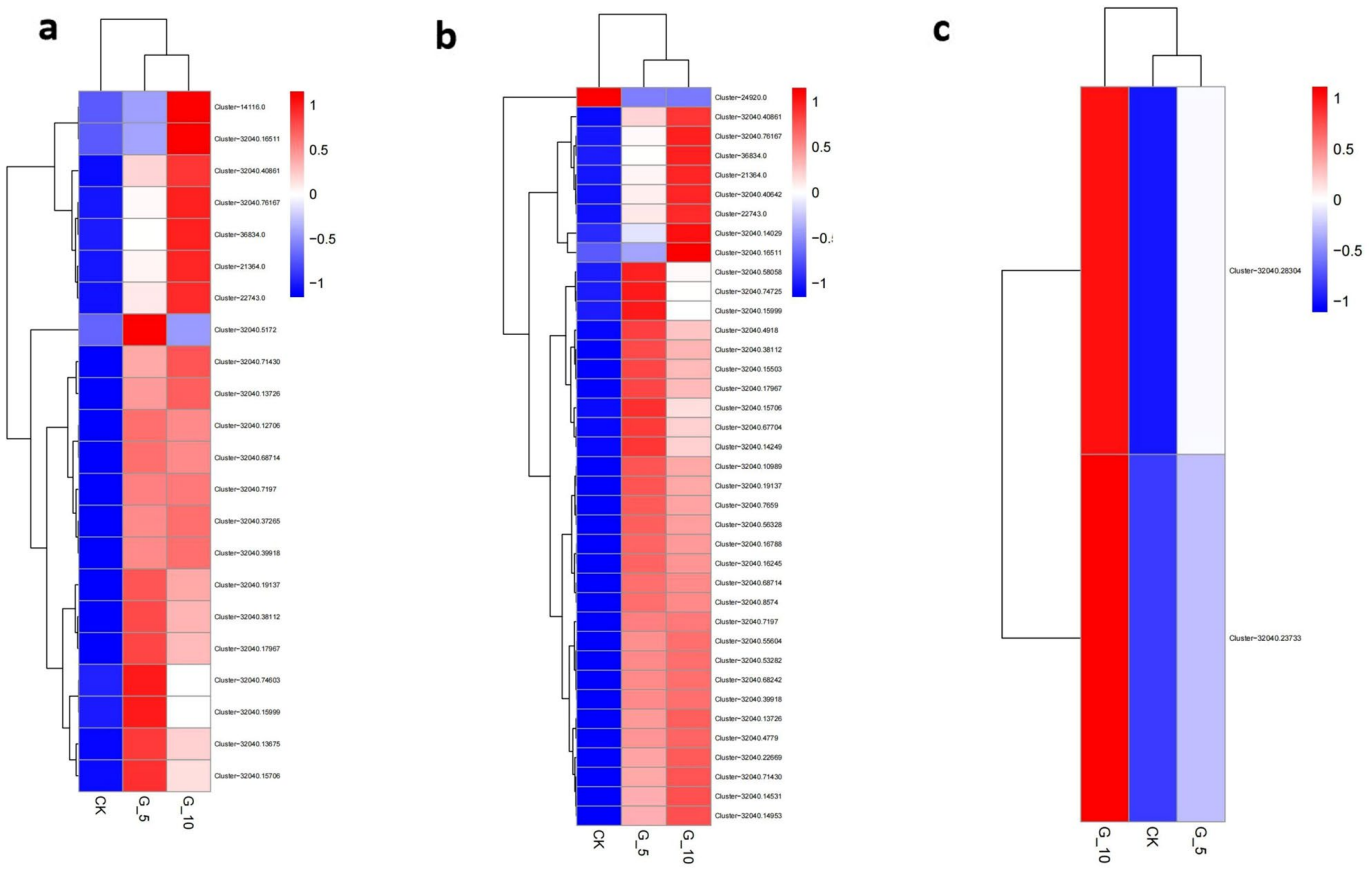
The unigenes involved in ME-IAA, IP, and ACC. (**a**) The expression of unigenes involved in ME-IAA. (**b**) The expression of unigenes involved in IP. (**c**) The expression of unigenes involved in ACC.

Plant hormones could regulate plant growth and development^11^. To further discover the genes involved in *B. striata* growth, we conducted an integrative analysis of hormone and transcriptome. Results showed that a total of 139 unigenes were suggested to be associated with 11 hormones, including ME-IAA, IP, tZ, DZ, H2JA, ABA, ACC, GA7, GA15, GA19, and GA20 (Table S4). Further, combined with the changing trend of hormone content, we found that ME-IAA, IP, and ACC were closely related to *B. striata* growth. Of those, 22, 38, and 2 unigenes were involved in ME-IAA, IP, and ACC, respectively (Fig. 8, Table S4). It was indicated that these unigenes were involved in *B. striata* growth.

## Discussions

Plants exhibited a variety of relationships with different fungi, such as symbiosis with mycorrhizal fungi or antagonistic associations with different pathogenic fungi in nature^12^. Endophytes colonizing healthy plant tissues at intercellular and/or intracellular levels exhibit a balanced antagonistic relationship with the host^13^. In recent years, endophyte resources, including fungal inoculation and the application of fungal elicitors, have been used in several biotechnology processes. Selective fungi and pathogens could promote the growth and development of plants and induce the accumulation of many types of active metabolites in the host^14–17^. A Fungal elicitor is a specific chemical signal derived from fungal cell extracts or secretions. In the interaction between plants and fungi, it could quickly, highly specifical, and selectively induce the expression of specific genes in plants, and then promote growth and the synthesis of secondary metabolites in plants. Lu^18^ screened out an endophytic fungus and made it into a fungal elicitor, and then found that it could significantly promote the growth and development of *Dendrobium officinale*. In another study, the fresh weight of *Dendrobium* treated by fungal elicitors was measured more than 4 times that of the control^19^. In our previous study, *Aspergillus flavus* was isolated from *B. striata*. In this study, we found that the *Aspergillus flavus* elicitor also could significantly promote the growth and development of *B. striata* (Fig. 1). It was consistent with the results mentioned above. Furthermore, as far as we knew, the *Aspergillus flavus* was isolated from *B. striata* for the first time and the genome information of *Aspergillus flavus* might be studied in the future.

Plant hormones are a kind of organic signal molecules produced by plant metabolism, which could influence physiological processes at very low concentrations^20^. Plant hormones were also referred to as “phytohormones” though this term was infrequently used. Plant hormones regulated various processes of plant growth, development, and environmental adaptation, which were independent of each other and cooperated to regulate vegetative growth, reproductive growth, seed germination, seed maturation and dormancy, embryonic development, as well as the adaptation to biotic and abiotic stresses in the growth cycle^11^. At present, the plant hormones including auxins, cytokinins, gibberellin, abscisic acid, ethylene, brassinosteroids, jasmonate, salicylic acid, and strigolactones have been studied deeply. To further study the possible relationship between hormones and *B. striata* growth, we performed the hormone metabonomics (Fig. 2). Results showed that ME-IAA, ICAld, SA, IP, and ACC might be involved in the growth of *B. striata*.

The plant hormone auxin was involved in the regulation of the growth and development in plants^21, 22^. Indole-3-acetic acid (IAA) was the first plant hormone identified in the plant hormone family and was the predominant form of auxin. Auxin signaling involved binding to F-box protein TIR1/AFB (TRANSPORT INHIBITOR RESPONSE/AUXIN SIGNALING F-BOX) together with a regulatory protein of the AUX/IAA family, forming a coreceptor complex. This resulted in ubiquitination and degradation of the AUX/IAA protein with the release of ARF (AUXIN TRANSCRIPTION FACTOR) transcription factors, leading to consequent changes in the expression of target genes. In the present study, 22 genes, including cluster 21,364.0, 32,040.13675, 14,116.0, 32,040.12706, 32,040.37265, 32,040.76167, 32,040.19137, 32,040.38112, 32,040.5172, 32,040.7197, 32,040.40861, 32,040.71430, 32,040.68714, 32,040.15706, 32,040.13726, 32,040.17967, 32,040.39918, 36,834.0, 22,743.0, 32,040.74603, 32,040.15999, and 32,040.16511 were involved in ME-IAA. For example, Cluster 32,040.37265 (Auxin transporter-like protein 2)^23^ and 14,116.0 (Transcription factor TCP13)^24^ had been proved to be beneficial for plant growth. It was indicated that these genes could be associated with *B. striata* growth and development.

Cytokinins (CKs) were plant hormones that could significantly regulate many aspects of plant development, including the morphogenesis and embryogenesis control, the initiation of cell division, and the delay in leaf senescence^25, 26^. Generally, biologically active cytokinins characterized a heterogeneous class of small, N6-substituted adenine derivatives with either an isoprene-derived or an aromatic side-chain^27^. The miscellaneous and explicit expression patterns of genes were found in cytokinin biosynthesis and metabolism, such as cytokinin biosynthesis isopentenyltransferase (IPT), cytokinin nucleoside 5-monophosphate phosphoribohydrolase (LOG), and cytokinin degrading cytokinin oxidase/dehydrogenase (CKX)^28, 29^. Less than a decade ago, for the first time, the transcription factor of CTK (ABCG14) was cloned from root to canopy^30, 31^. In the present study, a total of 28 genes were recommended to be involved in IP. Of those, cluster 32,040.19137 was annotated as ABC transporter G family member 11 (ABCG11) isoform X2, which is suggested to be intricated in the cytokinin translocation. Therefore, it was inferred that those genes may be involved in cytokinin metabolism, transport, and signal transduction, and the growth and development of *B. striata*.

Ethylene, a gaseous plant hormone by nature, performed a significant role in growth and development and facilitated to alleviate various biotic and abiotic stresses and pathogen infections in plants. Ethylene was synthesized from S-adenosylmethionine (SAM) via 1-aminocyclopropane-1-carboxylic acid (ACC). In plants, two vital enzymes in the ethylene biosynthetic pathway, namely ACC oxidase (ACO) and ACC synthase (ACS), strongly regulated both the transcriptional and post-transcriptional modulations in ethylene biosynthesis^32^. In our investigations, two genes, cluster 32,040.28304 (9-cis-epoxycarotenoid dioxygenase) and cluster 32,040.23733 were suggested to be involved in ACC. 9-cis-Epoxycarotenoid dioxygenase represented the key regulatory enzyme in the ABA biosynthesis pathway and played a significant role in ABA accumulation^33^. In the progression of growth and development in plants, ABA and ethylene not only collaborate with each other; however, also antagonized each other^34^. It was indicated that the signal pathways of various hormones might cross each other, forming complex regulatory networks, which affected the growth and development of *B. striata*.

## Conclusions

In this study, the *Aspergillus flavus* elicitor (1-G4) could significantly promote the growth and development of *B. striata*. The results of hormone determination showed that the content of SA, ICAld, IP, ACC, and ME-IAA were associated with the growth and development of *B. striata*. Transcriptomic analysis showed that a total of 1657 DEGs in CK Vs G5 and 2415 DEGs in CK vs G10 were identified. Furthermore, we found that 22, 28, and 2 unigenes were involved in ME-IAA, IP, and ACC, respectively. It was indicated that these unigenes might be involved in *B. striata* growth. The current study laid a theoretical foundation for the effective utilization of endophytic fungi and the optimization of germplasm resources of *B. striata*.

## Supplementary Information

Supplementary Information.
