# Supplementary material for: Integration of transcriptome and targeted metabolome profiling reveals hormone related genes involved in the growth of Bletilla striata
Source: Sci Rep. 2021 Nov 9;11:21950. doi: 10.1038/s41598-021-01532-8 (PMC8578652; doi:10.1038/s41598-021-01532-8)
Supplement: Supplementary file 1 — Supplementary Information. [file 41598_2021_1532_MOESM1_ESM.docx]

| Sample | Raw Reads | Clean Reads | Clean Bases | Error(%) | Q20(%) | Q30(%) | GC Content(%) |
| --- | --- | --- | --- | --- | --- | --- | --- |
| CK_1 | 45241634 | 44163862 | 6.62G | 0.03 | 97.94 | 93.91 | 46.81 |
| CK_2 | 47975638 | 46853486 | 7.03G | 0.02 | 98.06 | 94.19 | 46.69 |
| CK_3 | 48052798 | 46997176 | 7.05G | 0.02 | 98.07 | 94.2 | 46.67 |
| G_5_1 | 46722752 | 45690644 | 6.85G | 0.03 | 97.99 | 93.94 | 46.39 |
| G_5_2 | 47638504 | 46492938 | 6.97G | 0.03 | 97.97 | 93.94 | 46.38 |
| G_5_3 | 47143522 | 46003070 | 6.9G | 0.02 | 98.08 | 94.2 | 46.63 |
| G_10_1 | 47632678 | 46590274 | 6.99G | 0.02 | 98.13 | 94.29 | 46.39 |
| G_10_2 | 46945996 | 45995554 | 6.9G | 0.02 | 98.12 | 94.24 | 45.56 |
| G_10_3 | 47151452 | 46094172 | 6.91G | 0.02 | 98.07 | 94.14 | 46.13 |

**Table S1.** Offline data statistics

| Transcript length interval | 300-500bp | 500-1kbp | 1k-2kbp | >2kbp | Total |
| --- | --- | --- | --- | --- | --- |
| Number of transcripts | 58245 | 47084 | 40697 | 46977 | 193003 |
| Number of Unigenes | 35101 | 29307 | 16164 | 15085 | 95657 |

**Tables S2.** Transcripts and unigenes statistics and distribution

| Annotated in database | Number of Unigenes | Percentage (%) |
| --- | --- | --- |
| Annotated in NR | 41532 | 43.41 |
| Annotated in KEGG/KO | 9168 | 9.58 |
| Annotated in SwissProt | 23559 | 24.62 |
| Annotated in PFAM | 24382 | 25.48 |
| Annotated in GO | 24375 | 25.48 |
| Annotated in KOG/COG | 6027 | 6.3 |
| Total Unigenes | 95657 | 100 |

**Table S3.** Annotated statistics

| Gene Name | Meta Name | PCC | PCCP |
| --- | --- | --- | --- |
| Cluster-21364.0 | ME-IAA | 0.891 | 6.12e-8 |
| Cluster-32040.13675 | ME-IAA | 0.864 | 4.578e-7 |
| Cluster-14116.0 | ME-IAA | 0.808 | 0.000009302 |
| Cluster-32040.12706 | ME-IAA | 0.867 | 3.77e-7 |
| Cluster-32040.37265 | ME-IAA | 0.826 | 3.9477E-06 |
| Cluster-32040.76167 | ME-IAA | 0.898 | 3.36e-8 |
| Cluster-32040.19137 | ME-IAA | 0.807 | 9.9207E-06 |
| Cluster-32040.38112 | ME-IAA | 0.872 | 2.666e-7 |
| Cluster-32040.5172 | ME-IAA | 0.864 | 4.397e-7 |
| Cluster-32040.7197 | ME-IAA | 0.983 | 0 |
| Cluster-32040.40861 | ME-IAA | 0.814 | 7.1829E-06 |
| Cluster-32040.71430 | ME-IAA | 0.817 | 6.1272E-06 |
| Cluster-32040.68714 | ME-IAA | 0.801 | 1.29756E-05 |
| Cluster-32040.15706 | ME-IAA | 0.905 | 1.75e-8 |
| Cluster-32040.13726 | ME-IAA | 0.818 | 0.000005934 |
| Cluster-32040.17967 | ME-IAA | 0.823 | 4.6573E-06 |
| Cluster-32040.39918 | ME-IAA | 0.925 | 2e-9 |
| Cluster-36834.0 | ME-IAA | 0.878 | 1.7e-7 |
| Cluster-22743.0 | ME-IAA | 0.813 | 7.5098E-06 |
| Cluster-32040.74603 | ME-IAA | 0.805 | 1.08702E-05 |
| Cluster-32040.15999 | ME-IAA | 0.841 | 0.000001776 |
| Cluster-32040.16511 | ME-IAA | 0.982 | 0 |
| Cluster-21364.0 | IP | -0.85 | 1.0585E-06 |
| Cluster-32040.76167 | IP | -0.826 | 4.0772E-06 |
| Cluster-32040.55604 | IP | -0.815 | 6.6692E-06 |
| Cluster-32040.10989 | IP | -0.918 | 4.6e-9 |
| Cluster-32040.19137 | IP | -0.846 | 1.3313E-06 |
| Cluster-32040.38112 | IP | -0.831 | 3.1551E-06 |
| Cluster-32040.15503 | IP | -0.824 | 4.4368E-06 |
| Cluster-32040.7197 | IP | -0.843 | 1.5901E-06 |
| Cluster-32040.14029 | IP | -0.823 | 4.7344E-06 |
| Cluster-32040.40861 | IP | -0.808 | 9.6006E-06 |
| Cluster-32040.58058 | IP | -0.809 | 9.0946E-06 |
| Cluster-32040.71430 | IP | -0.807 | 9.7896E-06 |
| Cluster-32040.4918 | IP | -0.836 | 2.3091E-06 |
| Cluster-32040.68714 | IP | -0.836 | 2.3372E-06 |
| Cluster-32040.67704 | IP | -0.807 | 0.000009753 |
| Cluster-24920.0 | IP | 0.83 | 3.2272E-06 |
| Cluster-32040.15706 | IP | -0.83 | 3.3216E-06 |
| Cluster-32040.13726 | IP | -0.826 | 4.0385E-06 |
| Cluster-32040.17967 | IP | -0.81 | 8.6982E-06 |
| Cluster-32040.40642 | IP | -0.824 | 4.3422E-06 |
| Cluster-32040.68242 | IP | -0.802 | 1.21147E-05 |
| Cluster-32040.14531 | IP | -0.889 | 7.42e-8 |
| Cluster-32040.14953 | IP | -0.884 | 1.112e-7 |
| Cluster-32040.16788 | IP | -0.87 | 2.943e-7 |
| Cluster-32040.74725 | IP | -0.874 | 2.226e-7 |
| Cluster-32040.39918 | IP | -0.845 | 1.4631E-06 |
| Cluster-32040.22669 | IP | -0.853 | 8.912e-7 |
| Cluster-36834.0 | IP | -0.809 | 0.000009191 |
| Cluster-32040.53282 | IP | -0.839 | 2.0564E-06 |
| Cluster-22743.0 | IP | -0.827 | 3.8349E-06 |
| Cluster-32040.7659 | IP | -0.86 | 5.644e-7 |
| Cluster-32040.16245 | IP | -0.807 | 9.9304E-06 |
| Cluster-32040.8574 | IP | -0.81 | 8.4653E-06 |
| Cluster-32040.14249 | IP | -0.854 | 8.339e-7 |
| Cluster-32040.15999 | IP | -0.901 | 2.51e-8 |
| Cluster-32040.56328 | IP | -0.802 | 1.23985E-05 |
| Cluster-32040.16511 | IP | -0.832 | 2.8719E-06 |
| Cluster-32040.4779 | IP | -0.827 | 3.6928E-06 |
| Cluster-21364.0 | tZ | 0.887 | 8.32e-8 |
| Cluster-32040.13675 | tZ | 0.846 | 1.3493E-06 |
| Cluster-32040.12706 | tZ | 0.837 | 2.1932E-06 |
| Cluster-32040.76167 | tZ | 0.877 | 1.793e-7 |
| Cluster-32040.38112 | tZ | 0.863 | 4.653e-7 |
| Cluster-32040.5172 | tZ | 0.857 | 7.171e-7 |
| Cluster-32040.7197 | tZ | 0.967 | 0 |
| Cluster-32040.15706 | tZ | 0.895 | 4.18e-8 |
| Cluster-32040.13726 | tZ | 0.807 | 9.8221E-06 |
| Cluster-32040.39918 | tZ | 0.906 | 1.67e-8 |
| Cluster-36834.0 | tZ | 0.86 | 5.86e-7 |
| Cluster-22743.0 | tZ | 0.803 | 1.16262E-05 |
| Cluster-32040.15999 | tZ | 0.822 | 4.9193E-06 |
| Cluster-32040.16511 | tZ | 0.964 | 0 |
| Cluster-32040.21112 | DZ | -0.844 | 0.000001535 |
| Cluster-32040.39909 | DZ | -0.823 | 4.5547E-06 |
| Cluster-32040.48985 | DZ | -0.868 | 3.523e-7 |
| Cluster-32040.35897 | DZ | -0.983 | 0 |
| Cluster-32040.31407 | DZ | -0.809 | 9.0254E-06 |
| Cluster-32040.21737 | DZ | -0.816 | 6.5939E-06 |
| Cluster-32040.55859 | DZ | -0.908 | 1.36e-8 |
| Cluster-32040.21809 | DZ | -0.901 | 2.62e-8 |
| Cluster-32040.38112 | DZ | -0.907 | 1.42e-8 |
| Cluster-32040.7554 | DZ | -0.872 | 2.581e-7 |
| Cluster-32040.24059 | DZ | -0.895 | 4.23e-8 |
| Cluster-32040.28485 | DZ | -0.892 | 5.77e-8 |
| Cluster-32040.13626 | DZ | -0.836 | 2.3318E-06 |
| Cluster-32040.17966 | DZ | -0.808 | 9.3396E-06 |
| Cluster-32040.48229 | DZ | -0.889 | 7.44e-8 |
| Cluster-32040.67704 | DZ | -0.952 | 0 |
| Cluster-32040.44992 | DZ | -0.807 | 9.8513E-06 |
| Cluster-32040.19605 | DZ | -0.858 | 6.76e-7 |
| Cluster-32040.1483 | DZ | -0.826 | 0.000003895 |
| Cluster-32040.13726 | DZ | -0.81 | 8.6218E-06 |
| Cluster-32040.77757 | DZ | -0.818 | 0.000005884 |
| Cluster-32040.68242 | DZ | -0.807 | 9.6891E-06 |
| Cluster-32040.74725 | DZ | -0.913 | 7.7e-9 |
| Cluster-32040.47471 | DZ | -0.845 | 1.4462E-06 |
| Cluster-22743.0 | DZ | -0.807 | 9.8431E-06 |
| Cluster-32040.16245 | DZ | -0.815 | 6.8503E-06 |
| Cluster-32040.58058 | H2JA | -0.86 | 5.854e-7 |
| Cluster-32040.73178 | H2JA | -0.882 | 1.235e-7 |
| Cluster-32040.13435 | H2JA | -0.801 | 1.27369E-05 |
| Cluster-32040.37919 | H2JA | 0.823 | 4.5409E-06 |
| Cluster-32040.61122 | ABA | 0.852 | 9.429e-7 |
| Cluster-32040.23028 | ABA | 0.852 | 9.474e-7 |
| Cluster-32040.28304 | ACC | -0.81 | 8.7573E-06 |
| Cluster-32040.23733 | ACC | -0.879 | 1.628e-7 |
| Cluster-32040.5172 | GA7 | 0.804 | 1.13438E-05 |
| Cluster-32040.7197 | GA7 | 0.815 | 6.9506E-06 |
| Cluster-32040.15999 | GA7 | 0.819 | 0.000005699 |
| Cluster-21364.0 | GA15 | 0.854 | 8.406e-7 |
| Cluster-32040.13675 | GA15 | 0.834 | 2.6515E-06 |
| Cluster-32040.12706 | GA15 | 0.851 | 1.0299E-06 |
| Cluster-32040.76167 | GA15 | 0.876 | 1.936e-7 |
| Cluster-32040.38112 | GA15 | 0.867 | 3.642e-7 |
| Cluster-32040.5172 | GA15 | 0.867 | 3.766e-7 |
| Cluster-32040.7197 | GA15 | 0.971 | 0 |
| Cluster-32040.15706 | GA15 | 0.887 | 8.42e-8 |
| Cluster-32040.13726 | GA15 | 0.804 | 1.14811E-05 |
| Cluster-32040.39918 | GA15 | 0.895 | 4.41e-8 |
| Cluster-36834.0 | GA15 | 0.864 | 4.336e-7 |
| Cluster-32040.15999 | GA15 | 0.807 | 9.7573E-06 |
| Cluster-32040.16511 | GA15 | 0.967 | 0 |
| Cluster-21364.0 | GA19 | 0.849 | 1.1155E-06 |
| Cluster-32040.8501 | GA19 | 0.806 | 1.03041E-05 |
| Cluster-32040.22284 | GA19 | 0.804 | 1.11496E-05 |
| Cluster-35380.0 | GA19 | 0.853 | 8.997e-7 |
| Cluster-32040.67192 | GA19 | 0.823 | 4.5339E-06 |
| Cluster-29127.0 | GA19 | 0.809 | 8.9353E-06 |
| Cluster-32040.18206 | GA19 | 0.819 | 5.6764E-06 |
| Cluster-32040.39918 | GA19 | 0.803 | 1.17335E-05 |
| Cluster-32040.34828 | GA19 | 0.818 | 5.7735E-06 |
| Cluster-29275.0 | GA20 | -0.813 | 7.6157E-06 |
| Cluster-32040.26547 | GA20 | -0.8 | 1.33161E-05 |
| Cluster-32040.61122 | GA20 | -0.979 | 0 |
| Cluster-32040.54617 | GA20 | -0.802 | 1.23684E-05 |
| Cluster-32040.23028 | GA20 | -0.989 | 0 |
| Cluster-28756.0 | GA20 | -0.801 | 1.30142E-05 |

**Table S4.** The genes involved in hormones


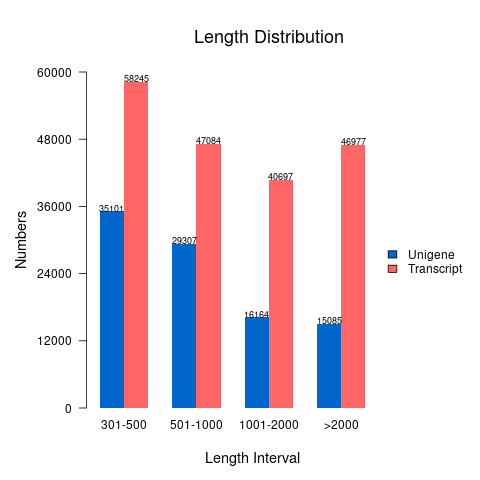


**Figure S1.** Distribution of the length interval of unigenes and transcript. X-axis represents the length interval; Y-axis represents the number of unigenes or transcripts: red pillars represent transcripts and blue pillars represent unigenes.


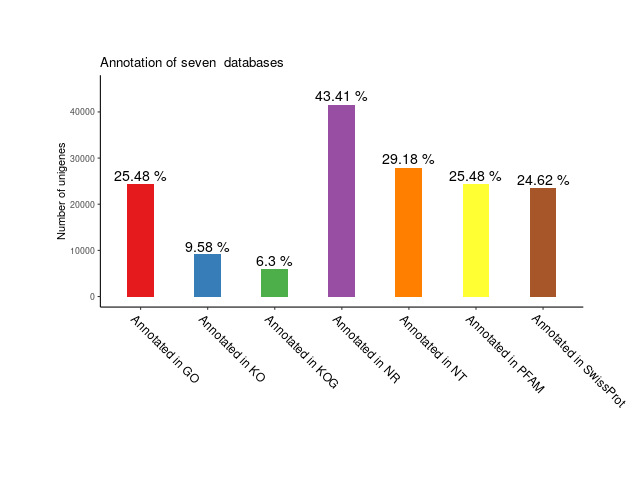


**Figure S2.** The unigenes annotated against seven databases. X-axis represents different database; Y-axis represents the number of unigenes.
